# Medication use by US patients with pulmonary hypertension associated with chronic obstructive pulmonary disease: a retrospective study of administrative data

**DOI:** 10.1186/s12890-022-02167-9

**Published:** 2022-10-18

**Authors:** Tracey J. Weiss, Dena Rosen Ramey, Lingfeng Yang, Xinyue Liu, Mahesh J. Patel, Swapnil Rajpathak, Ednan K. Bajwa, Dominik Lautsch

**Affiliations:** Merck & Co., Inc, Rahway, NJ USA

**Keywords:** Hypertension, Pulmonary hypertension, Retrospective study, Chronic obstructive pulmonary disease, Drug therapy, Algorithms

## Abstract

**Background:**

Pulmonary hypertension (PH) is a serious complication of chronic obstructive pulmonary disease (COPD). While clinical guidelines recommend specific drug therapies for pulmonary arterial hypertension (PAH), these drug therapies are not recommended for PH due to lung disease.

**Methods:**

This was a retrospective cohort study using the Optum® Clinformatics® Data Mart from January 2009–September 2019. An algorithm was designed to identify adults with ≥ 2 ICD-9-CM or ICD-10-CM diagnosis codes for PH and with ≥ 2 diagnosis codes for COPD. Sensitivity analyses were conducted among subgroups of patients with evidence of a right heart catheterization (RHC) or pulmonary function test (PFT). Patient characteristics, medications used, and durations of use of PAH and COPD medications were analyzed.

**Results:**

A total of 25,975 patients met the study inclusion criteria. Their mean age was 73.5 (SD 10.0) years and 63.8% were female. Medications targeting PAH were prescribed to 643 (2.5%) patients, most frequently a phosphodiesterase-5 inhibitor (2.1%) or an endothelin receptor antagonist (0.75%). Medications for COPD were prescribed to 17,765 (68.4%) patients, most frequently an inhaled corticosteroid (57.4%) or short-acting beta agonist (50.4%). The median durations of use ranged from 4.9 to 12.8 months for PAH medications, and from 0.4 to 5.9 months for COPD medications. Of the subgroup of patients with RHC (N = 2325), 257 (11.1%) were prescribed a PAH medication and 1670 (71.8%) used a COPD medication. Of the subgroup with a PFT (N = 2995), 58 (1.9%) were prescribed a PAH medication and 2100 (70.1%) a COPD medication.

**Conclusions:**

Patients with PH associated with COPD were identified in a US administrative claims database. Very few of these patients received any of the medications recommended for PAH, and only about two thirds received medications for COPD.

**Supplementary Information:**

The online version contains supplementary material available at 10.1186/s12890-022-02167-9.

## Background

Pulmonary hypertension (PH) is a pathophysiological disorder with involvement of the heart, lungs, and pulmonary vessels [1]. The common characteristic of this array of diseases is high blood pressure (defined as ≥ 20 mmHg) in the pulmonary arteries [2]. The World Health Organization (WHO) classifies PH into five distinct groups. PH due to chronic obstructive pulmonary disease (PH-COPD) is a subgroup of Group 3 (PH due to lung disease or hypoxia) [1]. The exact prevalence of PH in patients with COPD has not been determined, but appears to vary from 16 to 44% in mild COPD cases to 59–84% in severe cases [3].

The diagnosis of PH-COPD is difficult. COPD itself is diagnosed with pulmonary function tests using spirometry in patients with respiratory symptoms [4, 5]. However, there is no specific pattern of impairment of pulmonary function associated with the development of PH [6]. Non-invasive modalities—circulating biomarkers, echocardiography, and imaging—can screen for patients more likely to have PH associated with COPD [7]. Right-heart catheterization is the definitive test, but in practice is not commonly used to make this diagnosis. The European Society of Cardiology and the European Respiratory Society recommend against using right-heart catheterization to diagnose PH-COPD unless a therapeutic consequence is anticipated (lung transplantation, alternative PH diagnosis, or potential enrollment in a clinical trial) [1, 7]. Currently there are no guidelines published by US societies for the diagnosis and treatment of PH-COPD [7].

While the underlying COPD can be treated with inhaled bronchodilators and inhaled corticosteroids [4, 7], there are currently no medications for PH recommended or approved for PH-COPD [1]. Oxygen may be used, as needed. The pharmacotherapies recommended for pulmonary arterial hypertension (PAH) include endothelin receptor antagonists, phosphodiesterase type 5 inhibitors, and drugs targeting the prostacyclin pathway [1]. Certain patients also respond well to calcium channel blockers [8]. The efficacy of these pharmacotherapies in patients with PH-COPD has been evaluated in several randomized controlled trials [6, 7]. Meta-analysis of these trials indicates that targeted PAH therapy improved pulmonary hemodynamics but that the effects on symptoms and exercise capacity were inconsistent [9–11]. Therefore, current evidence is insufficient to support the use of PAH-targeted therapies in patients with PH-COPD [6, 7].

It is unclear how current guidelines translate into the use of PAH and COPD medications to treat PH-COPD in clinical practice in the United States. The objective of the present study was to describe the use of medications targeting either PAH or COPD using an administrative claims data set. Since there is currently no standard algorithm for identifying PH-COPD in administrative data, we first designed a diagnostic algorithm to achieve this goal.

## Methods

### Study design

This was a retrospective observational cohort study of administrative data in the Optum® Clinformatics® Data Mart for the period of January 2009–September 2019. The Optum® Data Mart is derived from a health insurance claims database of a geographically diverse population of members of large commercial and Medicare Advantage health plans. It contains de-identified data of plan members and their medical and pharmacy claims. In the current analysis, the annual number of enrolled adults ≥ 18 years of age between years 2014 and 2019 ranged from 12,913,087 to 17,364,906.

### Diagnostic algorithm

**Fig. 1:**
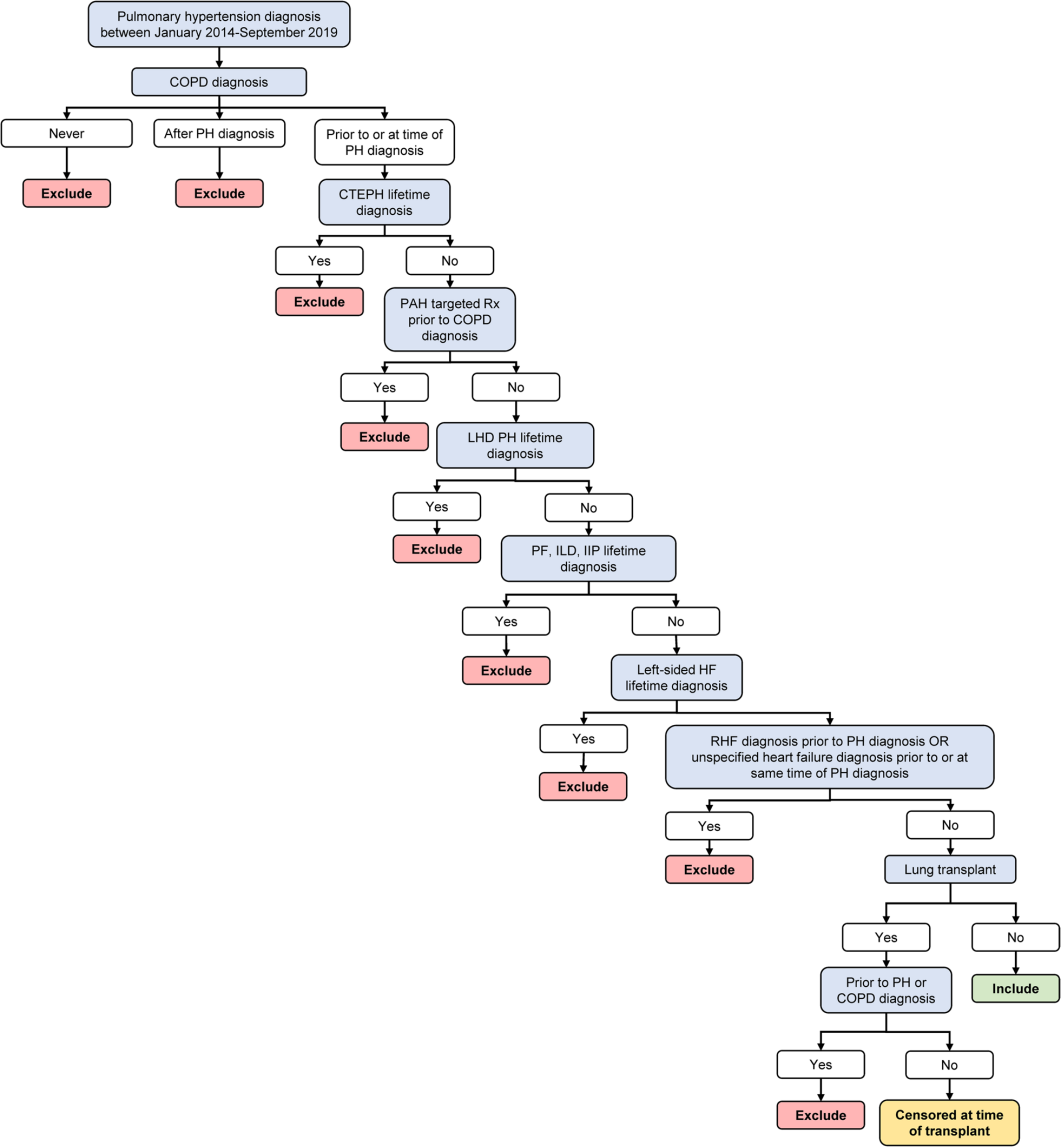
Algorithm to identify patients with pulmonary hypertension secondary to COPD. CTEPH, chronic thromboembolic pulmonary hypertension; COPD, chronic obstructive pulmonary disease; HF, heart failure; IIP, idiopathic interstitial pneumonia; ILD, interstitial lung disease; LHD, left heart disease; PAH, pulmonary arterial hypertension; PF, pulmonary fibrosis; PH, pulmonary hypertension

We designed an algorithm to identify PH-COPD patients in the Optum® Clinformatics® Data Mart, based on International Classification of Diseases, 9th edition clinical modification (ICD-9-CM), International Classification of Diseases, 10th edition clinical modification (ICD-10-CM), Current Procedural Terminology (CPT), and National Drug Codes (NDC) codes (Fig. 1). The algorithm required a diagnosis code for PH with a prior or concomitant diagnosis code for COPD and excluded patients with codes indicating WHO Group 3 lung diseases other than COPD, patients with a diagnosis code for WHO PH Group 4 (chronic thromboembolic pulmonary hypertension; CTEPH) or Group 2 (left-heart disease), and patients with NDC codes for Group 1 (PAH)-targeted medications occurring prior to the COPD diagnosis. The date of the first instance of a diagnosis code for PH in patients with at least two such codes was designated the index date, occurring within an index period from January 2014 to September 2019. Two or more codes for COPD were required, with the first preceding or coincident with the first code for PH.

### Study sample

Patients were included if they were ≥ 18 years old on the index date and had one of the following codes for PH: ICD-9-CM code 416.0 or ICD-10-CM codes I27.0, I27.2, I27.20, I27.21, I27.23, or I27.29 (Additional file 1: eTable 1). Patients also had to have a diagnosis of COPD. COPD was identified by codes for chronic bronchitis, emphysema, or other chronic airway obstruction: ICD-9-CM codes 491.x and ICD-10-CM codes J40, J41.x for chronic bronchitis; ICD-9-CM codes 492.x and ICD-10-CM codes J43.x for emphysema; ICD-9-CM code 496 for chronic airway obstruction and ICD-10-CM codes J44.x for COPD (Additional file 1: eTable 1).

Patients were excluded if they had ICD-10-CM codes 127.22 or 127.24, representing, respectively, PH due to left-heart disease, and CTEPH (Additional file 1: eTable 1). Patients were also excluded if they had codes for any of the following conditions: pulmonary fibrosis, interstitial lung disease, or idiopathic interstitial pneumonia (presumed to be other Group 3 PH diagnoses); left-heart failure, unspecified heart failure prior to the PH diagnosis, or right-heart failure prior to the PH diagnosis (presumed to be Group 2 PH); lung transplantation prior to the PH or COPD diagnosis. Finally, patients were excluded if they had either a diagnosis code for PH or an NDC code for a PAH-targeted therapy prior to the first diagnosis code for COPD, or if they died during the study period.

Two patient subgroups were defined for sensitivity analyses intended to increase diagnosis specificity: a subgroup with a right-heart catheterization (more specific for PH), and a subgroup with a pulmonary function spirometry test (more specific for COPD). A right-heart catheterization was identified by CPT codes 93451, 93453, 93456, 93457, 93460, 93461, 93530, 93531, 93532, or 93533 occurring prior to or up to 1 year after the index date. A pulmonary function test was identified by CPT codes 94010, 94060, 94375, 94200, 94726, 94727, 94728, 94016, 94729, or 95070 within the study period.

### Measurements

The presence of comorbidities and prescription fills for medicines targeting PAH and COPD were recorded. Comorbidities were recorded using all-available history prior to the index date. Within that period, patients had to have two records of a diagnosis code for a comorbidity: either two outpatient codes, or one outpatient and one inpatient code. PAH-targeted therapies were classified as endothelin receptor antagonists (bosentan, macitentan, and ambrisentan), PDE5 inhibitors (tadalafil and sildenafil), the soluble guanylate cyclase stimulator riociguat, and drugs acting on the prostacyclin pathway: the prostacyclin receptor agonist selexipag, and the prostacyclin analogs epoprostenol, treprostinil, and iloprost. COPD-targeted therapies consisted of inhaled short-acting beta agonists (albuterol, levalbuterol), short-acting anticholinergics (ipratropium), long-acting beta agonists (salmeterol, formoterol, arformoterol, aclidinium, and indacaterol), long-acting anticholinergic drugs (tiotropium), inhaled formulations of corticosteroids (budesonide, fluticasone, mometasone, beclomethasone, prednisone, prednisolone), and the PDE4 inhibitor roflumilast.

The duration of pharmacotherapy was measured from the time of the first prescription fill to the time of discontinuation. Discontinuation was defined as a period of ≥ 90 days with no record of a prescription after the end of days of supply of the last prescription fill. The time of discontinuation was defined as the first month after the end of days of supply. Patients lost to follow-up were censored.

### Data analysis

A descriptive analysis was conducted, with patient comorbidities and drug use presented as proportions. Durations of drug use were expressed as median, IQR and range.

## Results

### Patients

**Fig. 2:**
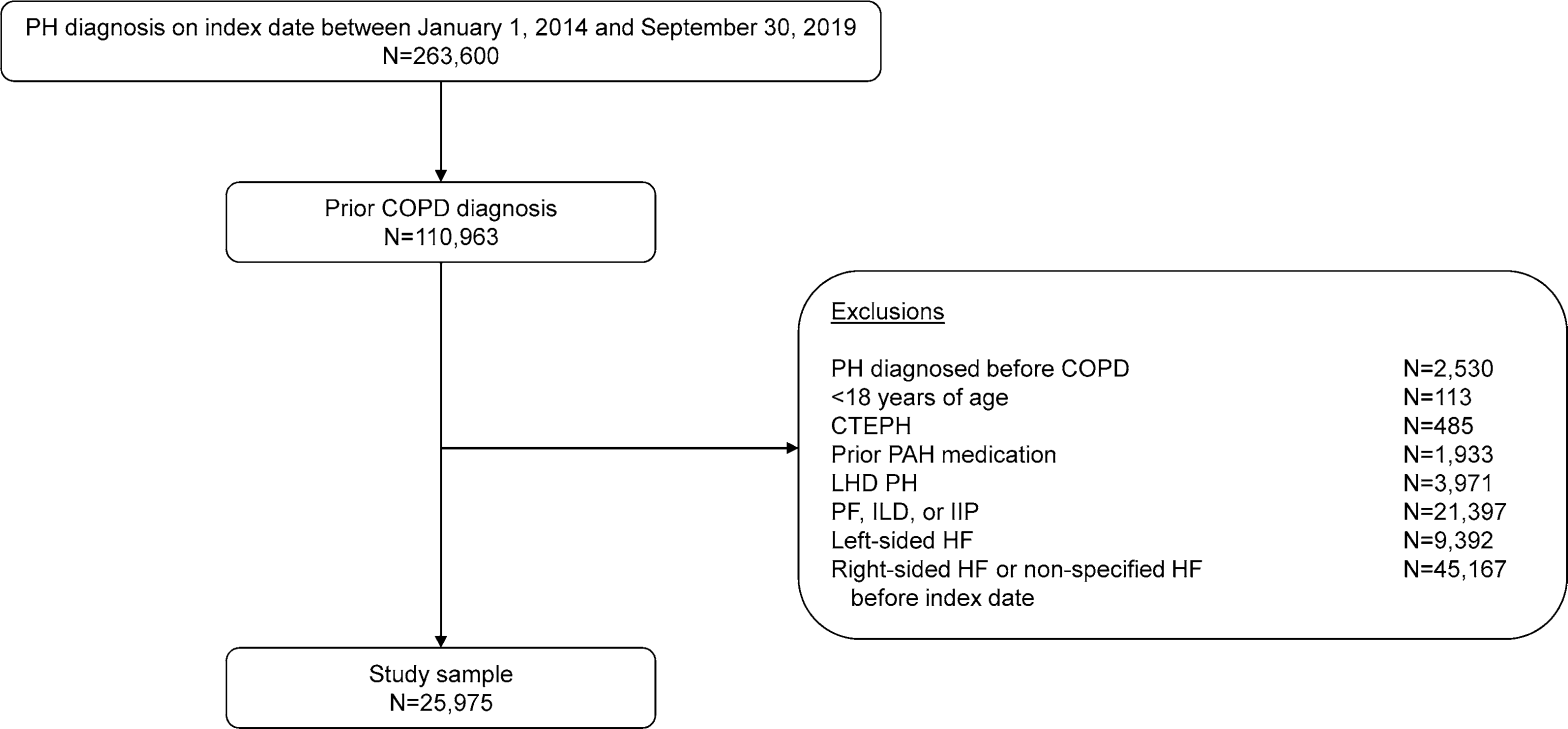
Selection of study population. CTEPH, chronic thromboembolic pulmonary hypertension; COPD, chronic obstructive pulmonary disease; HF, heart failure; IIP, idiopathic interstitial pneumonia; ILD, interstitial lung disease; LHD, left heart disease; PAH, pulmonary arterial hypertension; PF, pulmonary fibrosis; PH, pulmonary hypertension

A total of 263,600 adults had a PH diagnosis within the index period, of whom 110,963 (42.1%) had a prior COPD diagnosis, and 25,975 (9.9%) met the criteria for PH-COPD (Fig. 2). The median time between COPD and PH diagnoses was 13.8 months (range, 0–225 months). The mean patient age was 73.5 years, 63.8% were female, and the most frequent comorbidities were systemic hypertension (78.7%), asthma (64.7%), and coronary artery disease (42.7%; Table 1).

**Table 1:** Characteristics of PH-COPD patients and RHC and PFT subgroups

Characteristic	All COPD-PH (N = 25,975)	RHC (N = 2325)	PFT (N = 2995)
Age, mean (SD) years	73.5 (9.97)	70.6 (10.2)	73.5 (10.1)
Gender^a^			
Female	16,573 (63.8%)	1401 (60.3%)	2021 (67.5%)
Male	9399 (36.2%)	924 (39.7%)	974 (32.5%)
Comorbidities			
Hypertension (systemic)	20,448 (78.7%)	2050 (88.2%)	2238 (74.7%)
Asthma	16,806 (64.7%)	1757 (75.6%)	1792 (59.8%)
Heart disease			
Coronary artery disease	11,103 (42.7%)	1593 (68.5%)	1179 (39.4%)
Atrial fibrillation	8184 (31.5%)	859 (36.9%)	829 (27.7%)
Valvular heart disease	7359 (28.3%)	1161 (49.9%)	816 (27.2%)
Heart failure^b^	6974 (26.8%)	998 (42.9%)	624 (20.8%)
Myocardial infarction	4120 (15.9%)	486 (20.9%)	363 (12.1%)
Diabetes	8554 (32.9%)	910 (39.1%)	968 (32.3%)
Chronic kidney disease	7014 (27.0%)	720 (31.0%)	760 (25.4%)
Sleep apnea	6500 (25.0%)	831 (35.7%)	812 (27.1%)
Stroke	2137 (8.2%)	203 (8.7%)	257 (8.6%)
Connective tissue disease	806 (3.1%)	128 (5.5%)	136 (4.5%)

PFT, pulmonary function test; PH, pulmonary hypertension; RHC, right-heart catheterization; SD, standard deviation

^a^Gender identification was unavailable for 3 subjects in the PH-COPD group

^b^Right heart failure on or after the index date

### Medications for pulmonary arterial hypertension and COPD

Medications for PAH were used by 643 (2.5%) of the patient population, most frequently a PDE5 inhibitor (84.0%; Table 2). Of the 643 patients’ treatment courses, the most common was monotherapy (84.5%), followed by dual (18.4%) and triple (2.2%) therapy (note, a single patient could have a documented treatment course in one or more of these categories). The median durations of use ranged from 5.2 months for prostacyclin analogs to 10.8 months for endothelin receptor antagonists (Additional file 1: eTable 2).

**Table 2:** Medications for pulmonary arterial hypertension used by all PH-COPD patients and by RHC and PFT subgroups

Medication	All PH-COPD^a^ (N = 25,975)	RHC^a^ (N = 2325)	PFT^a^ (N = 2995)
Any PAH medication	643 (100%)	257 (100%)	58 (100.0)
Endothelin receptor antagonist	195 (30.3%)	106 (41.2%)	19 (32.8%)
PDE5 inhibitor	540 (84.0%)	214 (83.3%)	51 (87.9%)
Prostacyclin receptor agonist	30 (4.7%)	19 (7.4%)	5 (8.6%)
Prostacyclin analog	24 (3.7%)	13 (5.1%)	3 (5.2%)
Soluble guanylate cyclase stimulator	37 (5.8%)	20 (7.8%)	3 (5.2%)

PAH, pulmonary arterial hypertension; PFT, pulmonary function test; PH, pulmonary hypertension; RHC, right-heart catheterization

^a^The denominators for the percentages are the Ns with any PAH medication

**Fig. 3:**
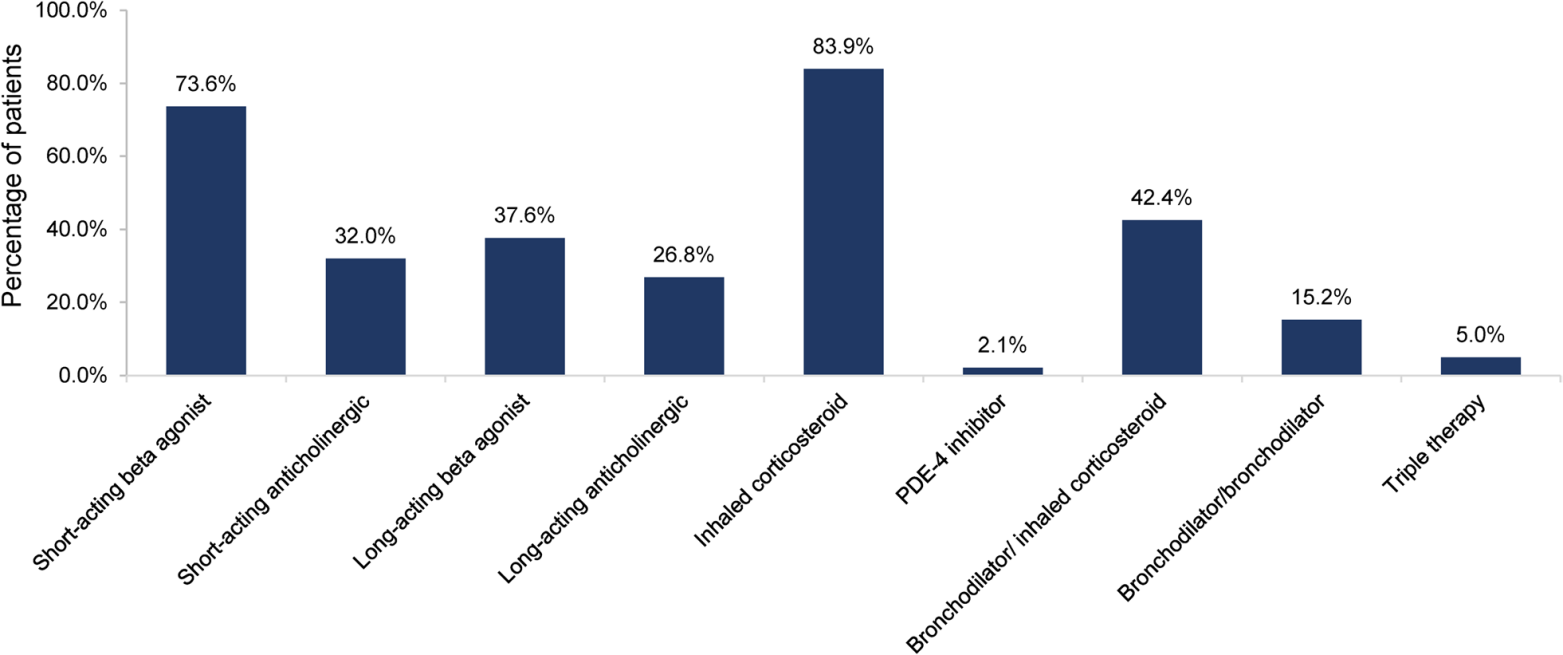
Medications for COPD used by all PH-COPD patients. COPD, chronic obstructive pulmonary disease. The denominators for the percentages are the Ns with any COPD medication (N = 17,765)

Medications for COPD were used by 17,765 (68.4%) of the study population. Most of these patients took an inhaled corticosteroid (83.9%) or short-acting beta agonist (73.6%; Table 3; Fig. 3). Inhaled bronchodilator/corticosteroid fixed-dose combinations were used by 42.4% of patients, bronchodilator/bronchodilator fixed-dose combinations by 15.2%, and the triple combination of fluticasone furoate/ umeclidinium/ vilanterol by 5.0% (Table 3; Fig. 3). The median durations of use ranged from 2.0 to 2.5 months for short-acting medications (anticholinergics or beta agonists) to 5.9 months for long-acting medications (anticholinergics, beta agonists, or bronchodilator/corticosteroid combinations; Additional file 1: eTable 3).

**Table 3:** Medications for COPD used by all PH-COPD patients and by RHC and PFT subgroups

Medication	All PH-COPD^a^ (N = 25,975)	RHC^a^ (N = 2325)	PFT^a^ (N = 2995)
Any COPD medication	17,765 (100%)	1670 (100%)	2100 (100%)
Short-acting beta agonist^b^	13,083 (73.6%)	1203 (72.0%)	1472 (70.1%)
Short-acting anticholinergic^b^	5692 (32.0%)	478 (28.6%)	516 (24.6%)
Long-acting beta agonist^b^	6674 (37.6%)	634 (38.0%)	761 (36.2%)
Long-acting anticholinergic^b^	4756 (26.8%)	448 (26.8%)	388 (18.5%)
Inhaled corticosteroid^b^	14,906 (83.9%)	1393 (83.4%)	1788 (85.1%)
PDE4 inhibitor	367 (2.1%)	35 (2.1%)	19 (0.9%)
Fixed-dose combination			
Bronchodilator/ inhaled corticosteroid	7530 (42.4%)	743 (44.5%)	884 (42.1%)
Bronchodilator/bronchodilator	2709 (15.2%)	290 (17.4%)	260 (12.4%)
Triple therapy^c^	880 (5.0%)	82 (4.9%)	58 (2.8%)

COPD, chronic obstructive pulmonary disease; PFT, pulmonary function test; PH, pulmonary hypertension; RHC, right-heart catheterization

^a^The denominators for the percentages are the Ns with any COPD medication

^b^Medication taken either alone or in a combination

^c^Fluticasone furoate/umeclidinium/vilanterol

### Subgroups with a right-heart catheterization or pulmonary function test

Of the 25,975 patients in the study population, 2325 (8.95%) had a right-heart catheterization. These patients were younger (mean age 70.6 years) and less predominantly female (60.3%) compared with the total study population (Table 1). All comorbidities were more prevalent than in the total study population, most notably asthma (75.6%), coronary artery disease (68.5%), valvular heart disease (49.9%), heart failure (42.9%), and sleep apnea (35.7%). Medications for PAH were used by 257 (11.1%) of this patient subset, most often a PDE5 inhibitor (83.3%; Table 2). Medications for COPD were used by 1670 (71.8%), most often an inhaled corticosteroid (83.4%) or short-acting beta agonist (72.0%; Table 3).

A pulmonary function test was recorded for 2995 patients, representing 11.5% of the study population. Demographics and comorbidities of the pulmonary function test subset were similar to those of the overall PH-COPD population (Table 1). Medications for PAH were used by 58 of these patients (1.9%) (Table 2) and medications for COPD by 2100 (70.1%). For both PAH and COPD drugs, the frequencies of use of the different drug classes were similar to those of the overall PH-COPD population (Table 3).

## Discussion

We designed an algorithm to identify PH-COPD in a health insurance data set, based on diagnostic, procedural, and pharmacy codes. The algorithm required a diagnosis code for PH with a prior or concomitant diagnosis code for COPD and excluded patients with codes indicating Group 3 lung diseases other than COPD and diagnosis codes for WHO Groups 4 (CTEPH) and 2 (left-heart disease), as well as patients with NDC codes for Group 1 (PAH)-targeted medications occurring prior to the COPD diagnosis. This algorithm identified a population that was on average 73.5 years of age and 63.8% female. Most PH-COPD patients (78.7%) had comorbid systemic hypertension and 64.7% had comorbid asthma. Consistent with The European Society of Cardiology and the European Respiratory Society guidelines that largely recommend against their use, very few (2.5%) PH-COPD patients received PAH medications; however, it was surprising that as many as 32.6% of patients had no documented medications to treat COPD given care guidelines call for optimization of COPD therapy in this population.

That said, the patterns of medication use by the PH-COPD patient set were consistent with the PH-COPD diagnosis. While the rate of use of medications to treat COPD was less than the 100% recommended in guidelines [1], it is comparable to rates in the range of 72–74% reported for adults with COPD in the United States [12, 13]. In an analysis of the US Medical Expenditure Panel Survey [14], 38.7% of adults with COPD used short-acting bronchodilators and 22.2% used bronchodilator/corticosteroid combinations—compared with 50.4% using short-acting beta agonists and 29.0% using bronchodilator/corticosteroid combinations in the present analysis. Low rates of adherence and/or persistence to medications by adults with COPD have been well documented [13, 15–17]. A median duration of use of inhaled therapies of 3.8 months has been reported for COPD patients [17]. These low rates and relatively short durations of drug treatment in patients with COPD or PH-COPD might represent either undertreatment or mild disease in many patients. The observation that the rate of pharmacotherapy is approximately the same in the subset of patients with a pulmonary function test provides some support for the former explanation.

The finding that fewer than 3% of PH-COPD patients used medications targeting PAH may not only reflect that practice aligns with care guidelines, but may also reflect an evolving understanding of the subset of patients with PH-COPD who may be better candidates for pharmacologic treatment with PH vasodilatory drugs: specifically, those with the pulmonary vascular phenotype, who comprise about 1–4% of patients with PH-COPD [7, 18, 19]. That is, the roughly 3% of patients reported in the present study could represent this patient subset being treated with off-label medications for PAH, though this cannot be confirmed in the present analysis. Alternatively, it is possible that some patients who have PAH and not PH-COPD were included in the study cohort. The presence of a small minority (2.2%) on triple therapy suggests that at least some patients were likely PAH patients as one would not expect a PH-COPD patient to be on triple therapy. Among the subgroup of patients with a right-heart catheterization, a group with an arguably higher probability of having PH-COPD, 89% of the PH-COPD patient set were not treated with PAH-targeted drugs. The 11% who did receive these drugs might indicate the off-label use of PAH medications in patients who are more likely to have PH-COPD. PH-COPD patients with a right-heart catheterization who were treated with PAH medications comprised only about 1% of the total study population.

Most patients were given a diagnosis of COPD without evidence of a pulmonary function test in the claims database. The 11.5% of patients who did have a record of a pulmonary function test had a similar rate of use of medications targeting COPD to that of the overall study population (70% versus 68%) and a similar demographic and comorbidity profile. The prevalence of asthma in this subgroup was slightly lower than in the overall population (59.8% versus 64.7%). (Note, the prevalence of asthma reported in the present study was higher than previously reported. Population-based estimates of the prevalence of asthma among patients with COPD alone are lower: 12%-29% in the United States, 17–18% in Spain, 23% in Latin America, and 28% in the United Kingdom [20–22]). Thus, there is no indication that this subset of patients had a more severe form of COPD, and it does not appear the addition of a pulmonary function test improved the specificity of the PH-COPD algorithm.

As discussed above, identifying PH-COPD is problematic. PH due to lung disease consists of several distinct diseases with similar symptoms [7]. Approximately 17% of patients with PAH also have COPD, and a clear distinction between PAH with concomitant COPD and PH due to COPD might not always be possible [7, 23]. While we attempted to account for this by requiring that the COPD diagnosis code precede or be coincident with the PH diagnosis, it is still possible we captured some COPD patients with concomitant PAH as opposed to PH-COPD patients. Further, right-heart catheterization, the definitive diagnostic test, is rarely performed in this population [7]. Identifying PH diagnoses in administrative data sets is especially challenging [24]. The ICD-9-CM code for PH does not distinguish between WHO PH Groups and, while there are ICD-10-CM codes specific to each WHO PH Group—e.g., ICD-10-CM I27.23 for Group 3—there is no code specific to PH-COPD. Medrek et al. identified PH-COPD using the combination of ICD-9-CM diagnosis codes for COPD and PH (respectively 491, 492, 494, 496 and 416.0, 416.8) in an analysis of the association of PH with more severe COPD in a Veterans Affairs data set [25]. However, systematic reviews of studies of the identification of PH in administrative data sets have concluded that algorithms relying solely on ICD diagnostic codes had low positive predictive value [24, 26, 27]. Algorithms for PAH that included procedural and drug codes for PAH-specific medications maximized the positive predictive value [24, 27]. Similarly, increasing algorithm complexity improved the positive predictive value of algorithms for CTEPH [27].

The limitations common to analyses of conditions using insurance claims data have been detailed previously [24]. These include a lack of information on disease severity, the absence of test results, potential errors or bias in coding, and turnover in enrollment. In the present study we used ICD-9-CM codes 491.x, 492.x, and 496 and ICD-10-CM codes J40, J41.x, J43.x, and J44.x to identify COPD. A systematic literature review of studies published between 2000 and 2018 found that ICD coding was the most commonly used variable to identify COPD patients [28]. The review noted that ICD-9-CM codes 490–496 and ICD-10-CM codes J41-J44 were used in 89% of the studies to identify COPD patients [28]. These codes roughly align with the codes used in the present study, however, there is a possibility that the codes used in the present study could inadvertently include patients with diseases other than COPD, as well as not capture all patients with COPD. In the case of claims-based analyses of PH, ICD-9-CM and ICD-10-CM do not align precisely with WHO clinical classifications, and the results of pulmonary function tests and right-heart catheterizations are not recorded. Hence, for example, patients might be classified as having PAH because of a right-heart catheterization test, yet not meet the hemodynamic criteria for PAH. In addition, some PAH-specific medications may be used off-label [24]. Further, because guidelines currently largely advise against RHC for PH-COPD, few patients had one documented, thus further limiting diagnosis specificity. The previously described methodology to use medications to increase positive predictive value of algorithms to identify other forms of PH (specifically PAH and CTEPH) cannot be applied to improve the positive predictive value for PH-COPD as there are no PH-COPD therapies currently approved. Thus, the present algorithm attempted to balance sensitivity, by using codes for non-specific PH, PAH, and Group 3 PH and temporally relating it to codes for COPD, and specificity, by removing codes specific to Group 2 and 4 PH and left heart failure. However, based on the results, it seems likely some misclassification and inclusion of patients with other forms of PH occurred. A validation study comparing the algorithm against a reference standard is needed to refine the algorithm to improve identification of PH-COPD patients and further evaluate medication use in this population.

## Conclusions

In this study we developed an algorithm to identify PH-COPD patients in a US commercial and Medicare Advantage claims data set. Most patients (two thirds) received medications recommended for COPD, which is a lower rate than expected from guidelines, while fewer than three percent of patients received medications targeting PAH, which is largely consistent with the absence of any recommended pharmacotherapy for PH in patients with PH-COPD.

## Supplementary Information

**Additional file 1.** Online supplement.** eTable 1**. ICD-9 and ICD-10 codes included or excluded from the algorithm.** eTable 2**. Duration of use of medications for pulmonary arterial hypertension in the PH-COPD population.** eTable 3**. Duration of use of medications for COPD in the PH-COPD population.

## Data Availability

The data that support the findings of this study are available from Optum® Clinformatics® Data Mart, but restrictions apply to the availability of these data, which were used under license for the current study, and so are not publicly available. Data are however available from the authors upon reasonable request and with permission of Optum®.

## Abbreviations

COPD: Chronic obstructive pulmonary disease
CPT: Current procedural terminology
CTEPH: Chronic thromboembolic pulmonary hypertension
ICD-9-CM: International Classification of Diseases, 9th edition Clinical Modification
ICD-10-CM: International Classification of Diseases, 10th edition Clinical Modification
NDC: National Drug Codes
PAH: Pulmonary arterial hypertension
PFT: Pulmonary function test
PH: Pulmonary hypertension
PH-COPD: Pulmonary hypertension due to chronic obstructive pulmonary disease
RHC: Right-heart catheterization
WHO: World Health Organization
