# Supplementary material for: Medication use by US patients with pulmonary hypertension associated with chronic obstructive pulmonary disease: a retrospective study of administrative data
Source: BMC Pulm Med. 2022 Oct 18;22:383. doi: 10.1186/s12890-022-02167-9 (PMC9578250; doi:10.1186/s12890-022-02167-9)
Supplement: Supplementary file 1 — Additional file 1. Online supplement. eTable 1. ICD-9 and ICD-10 codes included or excluded from the algorithm. eTable 2. Duration of use of medications for pulmonary arterial hypertension in the PH-COPD population. eTable 3. Duration of use of medications for COPD in the PH-COPD population. [file 12890_2022_2167_MOESM1_ESM.docx]

## Online supplement

eTable 1. ICD-9 and ICD-10 codes included or excluded from the algorithm

| Code | Condition | Included/Excluded |
| --- | --- | --- |
| **Pulmonary hypertension** | | |
| ICD-9 |  |  |
| 416.0 | Primary pulmonary hypertension | Included |
| ICD-10 |  |  |
| I27.0 | Primary pulmonary hypertension | Included |
| I27.2 | Other secondary pulmonary hypertension | Included |
| I27.20 | Pulmonary hypertension, unspecified | Included |
| I27.21 | Secondary pulmonary arterial hypertension | Included |
| I27.22 | Pulmonary hypertension due to left heart disease | Excluded |
| I27.23 | Pulmonary hypertension due to lung diseases and hypoxia | Included |
| I27.24 | Chronic thromboembolic pulmonary hypertension | Excluded |
| I27.29 | Other secondary pulmonary hypertension | Included |
| **COPD** | | |
| ICD-9 |  |  |
| 491 | Chronic bronchitis | Included |
| 491.0 | Simple chronic bronchitis | Included |
| 491.1 | Mucopurulent chronic bronchitis | Included |
| 491.2 | Obstructive chronic bronchitis | Included |
| 491.20 | Obstructive chronic bronchitis without exacerbation | Included |
| 491.21 | Obstructive chronic bronchitis with acute exacerbation | Included |
| 491.22 | Obstructive chronic bronchitis | Included |
| 491.8 | Other chronic bronchitis | Included |
| 491.9 | Unspecified chronic bronchitis | Included |
| 492 | Emphysema | Included |
| 492.0 | Emphysematous bleb | Included |
| 492.8 | Other emphysema | Included |
| 496 | Chronic airway obstruction, not elsewhere classified | Included |
| ICD-10 |  |  |
| J40 | Bronchitis, not specified as acute or chronic | Included |
| J41 | Simple and mucopurulent chronic bronchitis | Included |
| J41.0 | Simple chronic bronchitis | Included |
| J41.1 | Mucopurulent chronic bronchitis | Included |
| J41.8 | Mixed simple and mucopurulent chronic bronchitis | Included |
| J42 | Unspecified chronic bronchitis | Included |
| J43 | Emphysema | Included |
| J43.0 | Unilateral pulmonary emphysema (MacLeod’s syndrome) | Included |
| J43.1 | Panlobular emphysema | Included |
| J43.2 | Centrilobular emphysema | Included |
| J43.8 | Other emphysema | Included |
| J43.9 | Emphysema, unspecified | Included |
| J44 | Other chronic obstructive pulmonary disease | Included |
| J44.0 | Chronic obstructive pulmonary disease with (acute) lower respiratory infection | Included |
| J44.1 | Chronic obstructive pulmonary disease with (acute) exacerbation | Included |
| J44.9 | Chronic obstructive pulmonary disease, unspecified | Included |
| **Heart failure** | | |
| ICD-9 |  |  |
| 428.0 | Congestive heart failure, unspecified | * |
| 428.1 | Left heart failure | Exclude |
| 428.20 | Systolic heart failure, unspecified | * |
| 428.21 | Acute systolic heart failure | * |
| 428.22 | Chronic systolic heart failure | * |
| 428.23 | Acute on chronic heart failure | * |
| 428.30 | Diastolic heart failure, unspecified | * |
| 428.31 | Acute diastolic heart failure | * |
| 428.32 | Chronic diastolic heart failure | * |
| 428.33 | Acute on chronic diastolic heart failure | * |
| 428.40 | Combined systolic and diastolic heart failure, unspecified | * |
| 428.41 | Acute combined systolic and diastolic heart failure | * |
| 428.42 | Chronic combined systolic and diastolic heart failure | * |
| 428.43 | Acute on chronic combined systolic and diastolic heart failure | * |
| 428.9 | Heart failure, unspecified | * |
| ICD-10 |  |  |
| I50.1 | Left ventricular failure, unspecified | Exclude |
| I50.20 | Unspecified systolic (congestive) heart failure | * |
| I50.21 | Acute systolic (congestive) heart failure | * |
| I50.22 | Chronic systolic (congestive) heart failure | * |
| I50.23 | Acute on chronic systolic (congestive) heart failure | * |
| I50.30 | Unspecified diastolic (congestive) heart failure | * |
| I50.31 | Acute diastolic (congestive) heart failure | * |
| I50.32 | Chronic diastolic (congestive) heart failure | * |
| I50.33 | Acute on chronic diastolic (congestive) heart failure | * |
| I50.40 | Unspecified combined systolic (congestive) and diastolic (congestive) heart failure | * |
| I50.41 | Acute combined systolic (congestive) and diastolic (congestive) heart failure | * |
| I50.42 | Chronic combined systolic (congestive) and diastolic (congestive) heart failure | * |
| I50.43 | Acute on chronic combined systolic (congestive) and diastolic (congestive) heart failure | * |
| I50.810 | Right heart failure unspecified | ** |
| I50.811 | Acute right heart failure | ** |
| I50.812 | Chronic right heart failure | ** |
| I50.813 | Acute on chronic right heart failure | ** |
| I50.814 | Right heart failure due to left heart failure | Exclude |
| I50.82 | Biventricular heart failure | Exclude |
| I50.83 | High output heart failure | * |
| I50.84 | End stage heart failure | * |
| I50.89 | Other heart failure | * |
| I50.9 | Heart failure, unspecified | * |
| **Pulmonary fibrosis, interstitial lung disease, idiopathic interstitial pneumonia codes** | | |
| ICD-9 |  |  |
| 011.4 | Tuberculous fibrosis of lung | Exclude |
| 011.40 | Tuberculous lung fibrosis- unspecified | Exclude |
| 011.41 | Tuberculous lung fibrosis- no exam | Exclude |
| 011.42 | Tuberculous lung fibrosis- exam unknown | Exclude |
| 011.43 | Tuberculous lung fibrosis- micro diagnosis | Exclude |
| 011.44 | Tuberculous lung fibrosis- culture diagnosis | Exclude |
| 011.45 | Tuberculous lung fibrosis- history diagnosis | Exclude |
| 011.46 | Tuberculous lung fibrosis- other test | Exclude |
| 515 | Postinflammatory pulmonary fibrosis | Exclude |
| 516.3 | Idiopathic interstitial pneumonia | Exclude |
| 516.30 | Idiopathic interstitial pneumonia not otherwise specified | Exclude |
| 516.31 | Idiopathic pulmonary fibrosis | Exclude |
| 516.32 | Idiopathic non-spec interstitial pneumonia | Exclude |
| 516.33 | Acute interstitial pneumonia | Exclude |
| 516.35 | Idiopathic lymphocytic interstitial pneumonia | Exclude |
| 516.36 | Cryptogenic organizing pneumonia | Exclude |
| 516.37 | Desquamative interstitial pneumonia | Exclude |
| ICD-10 |  |  |
| J84.1 | Other interstitial pulmonary diseases with fibrosis | Exclude |
| J84.10 | Pulmonary fibrosis, unspecified | Exclude |
| J84.11 | Idiopathic interstitial pneumonia | Exclude |
| J84.111 | Idiopathic interstitial pneumonia, not otherwise specified | Exclude |
| J84.112 | Idiopathic pulmonary fibrosis | Exclude |
| J84.113 | Idiopathic non-specific interstitial pneumonitis | Exclude |
| J84.114 | Acute interstitial pneumonitis | Exclude |
| J84.115 | Respiratory bronchiolitis interstitial lung disease | Exclude |
| J84.116 | Cryptogenic organizing pneumonia | Exclude |
| J84.117 | Desquamative interstitial pneumonia | Exclude |
| J84.17 | Other interstitial pulmonary disease with fibrosis in disease classified elsewhere | Exclude |
| J84.2 | Lymphoid interstitial pneumonia | Exclude |

PH-COPD, chronic obstructive pulmonary disease-pulmonary hypertension

* Exclude if prior to or at the same time as PH diagnosis (index date). Include if after PH diagnosis.

** Exclude if prior to PH diagnosis (index date). Include if at the same time or after the PH diagnosis.

eTable 2. Duration of use of medications for pulmonary arterial hypertension in the PH-COPD population

| Medication | N (%) ^*^ | Duration of use (Months) | |
| --- | --- | --- | --- |
|  |  | Median | Range |
| Endothelin receptor antagonist | 195 (30.3%) | 10.8 | (0.99, 68.8) |
| Bosentan | 19 (3.0%) | 12.8 | (0.99, 67.9) |
| Macitentan | 86 (13.4%) | 10.4 | (0.99, 56.2) |
| Ambrisentan | 100 (15.6%) | 10.4 | (0.99, 68.8) |
| PDE5 inhibitor | 540 (84.0%) | 6.9 | (0.10, 68.8) |
| Tadalafil | 193 (30.0%) | 8.9 | (0.10, 68.8) |
| Sildenafil | 360 (56.0%) | 5.9 | (0.23, 66.8) |
| Prostacyclin receptor agonist | 30 (4.7%) | 6.3 | (0.92, 41.0) |
| Selexipag | 30 (4.7%) | 6.3 | (0.92, 41.0) |
| Prostacyclin analog | 24 (3.7%) | 5.2 | (0.92, 33.5) |
| Epoprostenol | 1 (0.16%) | 4.9 | (4.93, 4.9) |
| Treprostinil | 23 (3.6%) | 5.5 | (0.92, 33.5) |
| Iloprost | 0 (-) | - | - |
| Soluble guanylate cyclase stimulator | 37 (5.8%) | 6.9 | (0.46, 55.2) |
| Riociguat | 37 (5.8%) | 6.9 | (0.46, 55.2) |

PH-COPD, chronic obstructive pulmonary disease-pulmonary hypertension; PAH, pulmonary arterial hypertension.

^*^ Percent of N=643.

eTable 3. Duration of use of medications for COPD in the PH-COPD population

| Medication | N (%) ^*^ | Duration of use (Months) † | |
| --- | --- | --- | --- |
|  |  | Median | Range |
| Short-acting beta agonist | 13,083 (73.6%) | 2.5 | (0.03, 68.9) |
| Albuterol | 12,882 (72.5%) | 2.5 | (0.03, 68.9) |
| Levalbuterol | 564 (3.2%) | 1.2 | (0.07, 63.5) |
| Short-acting anticholinergic | 5,692 (32.0%) | 2.0 | (0.03, 68.5) |
| Ipratropium | 5,692 (32.0%) | 2.0 | (0.03, 68.5) |
| Long-acting beta agonist | 6,674 (37.6%) | 5.9 | (0.03, 68.3) |
| Salmeterol | 3,111 (17.5%) | 4.9 | (0.03, 59.1) |
| Formoterol | 3,744 (21.1%) | 4.9 | (0.03, 68.3) |
| Arformoterol | 246 (1.4%) | 2.5 | (0.03, 56.2) |
| Aclidinium | 122 (0.69%) | 6.0 | (0.03, 50.3) |
| Indacaterol | 8 (0.05%) | 5.9 | (0.99, 32.5) |
| Long-acting anticholinergic | 4,756 (26.8%) | 5.9 | (0.03, 68.3) |
| Tiotropium | 4,756 (26.8%) | 5.9 | (0.03, 68.3) |
| Corticosteroid | 14,906 (83.9%) | 3.1 | (0.03, 68.9) |
| Budesonide | 4,130 (23.2%) | 3.9 | (0.03, 68.3) |
| Fluticasone | 5,884 (33.1%) | 4.9 | (0.03, 67.6) |
| Mometasone | 305 (1.7%) | 3.0 | (0.03, 39.4) |
| Beclomethasone | 130 (0.73%) | 3.0 | (0.66, 53.2) |
| Prednisone | 10,044 (56.5%) | 0.6 | (0.03, 68.9) |
| Prednisolone | 5,187 (29.2%) | 0.4 | (0.03, 44.4) |
| PDE4 inhibitor | 367 (2.1%) | 5.9 | (0.46, 66.1) |
| Roflumilast | 367 (2.1%) | 5.9 | (0.46, 66.1) |
| Bronchodilator/ corticosteroid combination | 7,530 (42.4%) | 5.9 | (0.03, 68.3) |
| Budesonide/formoterol fumarate | 3,260 (18.4%) | 4.9 | (0.03, 68.3) |
| Fluticasone propionate/salmeterol | 2,995 (16.9%) | 4.9 | (0.03, 59.1) |
| Fluticasone/vilanterol | 1,908 (10.7%) | 3.9 | (0.46, 47.3) |
| Mometasone/formoterol | 254 (1.4%) | 3.0 | (0.03, 39.4) |
| Bronchodilator combination | 2,709 (15.2%) | 3.9 | (0.03, 67.8) |
| Aclidinium bromide/formoterol fumarate | 0 (-) | - | - |
| Ipratropium/albuterol sulfate | 933 (5.3%) | 3.0 | (0.03, 67.8) |
| Glycopyrrolate/formoterol fumarate | 168 (0.95%) | 3.0 | (0.49, 31.5) |
| Indacaterol/glycopyrrolate | 2 (0.01%) | 1.0 | (0.99, 0.99) |
| Tiotropium bromide/olodaterol HCl | 531 (3.0%) | 3.9 | (0.99, 48.3) |
| Umeclidinium bromide/vilanterol trifenatate | 1,290 (7.3%) | 3.9 | (0.23, 58.2) |
| Triple therapy | 880 (5.0%) | 3.0 | (0.46, 21.7) |
| Fluticasone/umeclidinium/vilanterol | 880 (5.0%) | 3.0 | (0.46, 21.7) |

COPD, Chronic obstructive pulmonary disease

^*^ Percent of N=17,765.

^†^ Numbers are median (minimum, maximum).
